# Percutaneous cryoablation versus robot-assisted partial nephrectomy for small renal cell carcinoma: a retrospective cost analysis at Japanese single-institution

**DOI:** 10.1007/s10147-025-02783-5

**Published:** 2025-06-06

**Authors:** Mayu Uka, Toshihiro Iguchi, Kensuke Bekku, Tomoaki Yamanoi, Toshiharu Mitsuhashi, Hideo Gobara, Noriyuki Umakoshi, Takahiro Kawabata, Koji Tomita, Yusuke Matsui, Motoo Araki, Takao Hiraki

**Affiliations:** 1 Department of Radiology, Okayama University Hospital, Okayama, Japan; 2 Department of Radiology, NHO Okayama Medical Center, Okayama, Japan; 3 Department of Radiological Technology, Faculty of Health Sciences, Okayama University, Okayama, Japan; 4 Department of Urology, Okayama University Graduate School of Medicine, Dentistry, and Pharmaceutical Sciences, Okayama, Japan; 5 Center for Innovative Clinical Medicine, Okayama University Hospital, Okayama, Japan; 6 Division of Medical Informatics, Okayama University Hospital, Okayama, Japan; 7 Department of Radiology, Faculty of Medicine, Dentistry and Pharmaceutical Sciences, Okayama University, Okayama, Japan

**Keywords:** Renal cancer, Cryoablation, Robot-assisted partial nephrectomy, Cost, Cost effectiveness

## Abstract

**Background:**

No direct cost comparison has been conducted between percutaneous cryoablation (PCA) and robot-assisted partial nephrectomy (RAPN) for clinical T1a renal cell carcinoma (RCC) in Japan. This study aimed to compare their costs.

**Methods:**

We retrospectively analyzed data from 212 PCAs (including 155 with transcatheter arterial embolization) and 119 RAPN cases performed between December 2017 and May 2022.

**Results:**

PCA patients were older with higher American Society of Anesthesiologists scores, Charlson Comorbidity Index, and history of previous RCC treatment, cardiovascular disease, and antithrombotic drug use than RAPN patients. PCA was associated with a significantly shorter procedure time and hospitalization duration with fewer major complications than those associated with RAPN. While PCA incurred a slightly lower total cost (1,123,000 vs. 1,155,000 yen), it had a significantly higher procedural cost (739,000 vs. 693,000 yen) and markedly worse total (− 93,000 vs. 249,000 yen) and procedural income-expenditure balance (− 189,000 vs. 231,000 yen) than those of RAPN. After statistical adjustment, PCA demonstrated significantly higher total (difference: 114,000 yen) and procedural costs (difference: 72,000 yen), alongside significantly worse total (difference: − 358,000 yen) and procedural income-expenditure balances (difference: − 439,000 yen). The incremental cost-effectiveness ratio was more favorable for PCA than for RAPN.

**Conclusion:**

For high- risk patients, PCA demonstrated a safer option with shorter hospitalization duration than those of RAPN. Although PCA was more cost-effective, its higher procedural cost and unfavorable income-expenditure balance require careful evaluation, especially for large tumors that require three or more needles.

## Introduction

The incidence of renal cell carcinoma (RCC) has increased worldwide, including in Japan, due to advancements in diagnostic imaging and increased screening [1]. The most incidentally detected RCCs are small (< 4 cm; T1a), asymptomatic, and localized (N0, M0). Although partial nephrectomy is the standard treatment for small RCC, alternative options such as ablation therapy and active surveillance are also considered based on patient-specific factors, including age, renal function, and comorbidities [2, 3].

Robot-assisted laparoscopic partial nephrectomy (RAPN), which has been covered by national insurance since 2016, is a minimally invasive nephron-sparing procedure that preserves renal function better than radical nephrectomy [4] and reduces complications compared to open partial nephrectomy [5]. In contrast, percutaneous cryoablation (PCA), which has been covered by national insurance since 2011, is often performed for patients unsuitable for surgery. PCA is particularly effective for small RCCs, with a high success rate (> 90% local control and nearly 100% 5 year survival) and few major complications [6–9]. Several studies have compared these two treatments in terms of local control, survival, renal function, complications, and hospitalization [10–14].

Given the increasing healthcare costs associated with Japan’s aging population, cost comparisons among treatment options are crucial. Although previous studies have compared the costs of PCA and RAPN [15–17], no cost analysis has been conducted in Japan. The results of this unique Japanese evaluation may contribute to more cost-effective treatment strategies for small RCC in the Japanese healthcare system. This retrospective study compared the costs of PCA and RAPN at a single Japanese institution.

## Materials and methods

Our ethics committee approved this study (approval number: KEN2211-029) and waived the need for informed consent due to its retrospective nature. However, opt-out consent was obtained for patients’ data use, and written informed consent was secured before treatment.

### Patients

We included patients who underwent PCA or RAPN as initial treatment for T1a RCC between December 2017 and May 2022. Exclusion criteria were: (i) renal tumor biopsy or angiography without transcatheter arterial embolization (TAE) during the same hospitalization; (ii) PCA for multiple RCCs in one admission; (iii) treatment for other diseases during the same hospitalization; (iv) heparinization before RCC treatment; (v) metastatic lesions; (vi) recurrent RCC; (vii) enrollment in clinical trials; or (viii) insufficient data. Treatment strategies were decided at multidisciplinary team conferences, considering comorbidities, renal function, and patient preferences. PCA was used for patients unsuitable for surgery due to old age, comorbidities, or refusal.

### Study endpoint

The primary endpoint was the cost of PCA and RAPN. Secondary endpoints included procedure time, hospitalization duration, safety (major complications), and incremental cost-effectiveness (ICER). Costs were assessed from a hospital perspective. Complications were graded per the Clavien–Dindo classification [18], with major complications defined as grade III or higher.

### Transarterial embolization before PCA

Most PCA patients underwent TAE 1–3 days before ablation by interventional radiologists during the same hospitalization. TAE enhances RCC visualization under CT-fluoroscopy, minimizes the heat sink effect, and reduces bleeding and seeding risks. Indications for TAE were determined by consensus; it was not performed when tumors were easily identified on plain CT, exhibited poor vascularity, or in patients unable to use contrast media due to severe renal impairment or iodine allergy.

Under local anesthesia, a 4 F catheter was inserted via the common femoral artery. After selecting the renal artery, angiography was performed to identify tumor-supplying vessels. Tumor-feeding arteries were selectively catheterized using a 1.5–2.7 F microcatheter. Embolization was performed with iodized oil and absolute ethanol or a gelatin sponge, with coils used when necessary.

### PCA

PCA was performed using an argon-based cryoablation system (CryoHit^®^ or VISUAL ICE™, Boston Scientific, Marlborough, MA) with 17-gauge cryoprobes (IceRod™, IceSeed™, IceRod 1.5 Plus™, or Ice Sphere™, Boston Scientific) under local or general anesthesia and CT-fluoroscopy guidance in an interventional radiology suite. For RCC near vital organs such as the colon, hydrodissection was achieved by infusing a saline-2% contrast mixture to create a safe distance. The number of cryoprobes was decided by consensus. Typically, three were used for RCCs > 15 mm, and two for small RCCs.

After insertion, cryoablation was performed in two 10–15 min freezing cycles, separated by ≥ 2 min of passive thawing. After each cycle, CT imaging confirmed that the RCC was fully contained within the ice ball, ensuring an ablation margin of at least 6 mm [19]. If coverage was incomplete, additional freeze–thaw cycles were performed after repositioning the cryoprobes.

### RAPN

RAPN was performed by urologists under general anesthesia using the da Vinci Surgical System Si and Xi (Intuitive Surgical, Sunnyvale, CA, USA). A transperitoneal or retroperitoneal approach was chosen based on tumor location, size, and surgical history. Tumors were typically resected with total renal artery clamping under warm ischemia. Pyelopelvic suturing was performed with the urinary tract open; parenchymal sutures and a hemostat were used before cortical closure in most cases.

### Cost data collection

Cost terms were defined as in Table 1, representing “costs from the hospital perspective”. Total treatment cost was calculated by adding non-reimbursable supplies and medications (e.g., cryoprobes, gas, surgical instruments, disposables, iodized oil, and contrast media for hydrodissection); reimbursable supplies, medications, and transfusion costs (e.g., catheters and contrast media for TAE and other medication during hospitalization); depreciation of surgical and interventional radiology suites (including robotic and cryoablation systems and a unified CT and angiography system); and anesthesia equipment. Personnel costs for the operator, circulating nurses, surgical technologists, and radiological technologists were included. Total income (the hospital payment for each treatment) was calculated by adding procedure fees (based on insurance points), anesthesia fees, other fees (e.g., for transfusions and ICU admissions), reimbursable supplies and medications, and hospital income based on the Diagnosis Procedure Combination (DPC)/Per-Diem Payment Systems (PDPS). The hospital used activity-based costing, reflecting allocation based on specific medical procedures and resource usage.

**Table 1:** Definitions of cost terms

Term	Definition
Total income	Total income during hospitalization per case
Total cost	Total expenditure during hospitalization
Total income-expenditure balance	Income and expenditure during hospitalization
Procedural income	Income from one surgical procedure (PCA or RAPN)
Procedural cost	Expenditure per surgical procedure (PCA or RAPN)
procedural income–expenditure balance	Income and expenditure per surgical procedure (PCA or RAPN)

*PCA* percutaneous cryoablation, *RAPN* robot-assisted partial nephrectomy

Data on cryoprobes, catheters, surgical instruments, and procedure time were obtained from surgical records and the Data Warehouse. Gas usage (argon and helium) was sourced from the Medical Engineer Center database (one or two bottles of argon and 0.2 bottles of helium per case). Medications, transfusions, and anesthesia eligible for insurance claims were extracted from the DPC database. Treatment income was calculated per Japanese National Insurance as follows: TAE (code K-6153), 186,200 yen; PCA (code K773-4) at 528,000 yen; and RAPN (code K773-5) at 707,300 yen. Depreciation and labor costs were calculated based on cost price, frequency of use, and procedure time. Pathology fees for RAPN were excluded since biopsies for pathologic diagnosis before PCA were performed during separate admissions, thus excluded from the cost analysis.

### Cost-effectiveness analysis

Effectiveness was evaluated based on the frequency of complications, following a previous report [16]. Since the complication rate was low in this study, the ICER was calculated using the rates reported in a systematic review [20].

### Statistical analysis

Data on patient age, sex, body mass index, comorbidities (diabetes, hypertension, and cardiovascular disease [CVD]), prior RCC treatment, single kidney status, antithrombotic drug use, tumor size, and R.E.N.A.L. nephrometry score were collected. Patients were classified according to ASA score and Charlson Comorbidity Index (CCI). These factors were statistically compared between the groups.

Descriptive statistics were used to summarize continuous variables (e.g., means and standard deviations) and categorical variables (e.g., frequencies and proportions). Outcomes were compared among three groups: PCA without TAE, PCA with TAE, and RAPN. These outcomes included total income, total cost, income-expenditure balance, procedural income, procedural cost, procedural income-expenditure balance, hospitalization  duration, and procedure time. The costs of PCA with and without TAE were compared to those of RAPN, and the differences and 95% confidence intervals were calculated.

To adjust for potential confounders, we used inverse probability weighted regression adjustment (IPWRA), which combines score weighting and regression adjustment to reduce bias. This method accounts for baseline differences and provides treatment effect estimates similar to those from randomized controlled trials. We used a treatment model to estimate the probability of each patient receiving a particular treatment, based on the covariates listed in Table 2. An outcome model was then used to estimate the outcomes while adjusting for these covariates, as well as single kidney status and hereditary renal cancer. By combining the two models, the IPWRA achieves double robustness, meaning that correct estimates can be obtained even if one of the models is misspecified [21].

**Table 2:** Patient and tumor demographics

			SMD		VR	
Parameter	PCA (n = 212)	RAPN (n = 119)	Raw	Weighted	Raw	Weighted
Age (years), mean (SD)	65.1 (13.7)	62.9 (9.9)	0.19	− 0.19	1.88	2.75
Male sex, n (%)	157 (74.1)	81 (68.1)	0.13	− 0.23	0.88	1.38
Body mass index (kg/m^2^), mean (SD)	23.8 (4.0)	24.7 (3.4)	− 0.22	− 0.14	1.44	2.63
ASA score, mean (SD)	2.2 (0.6)	1.8 (0.5)	0.57	− 0.40	1.46	0.82
CCI, mean (SD)	3.5 (2.0)	2.7 (1.5)	0.47	− 0.42	1.65	1.09
Hypertension, n (%)	128 (60.4)	59 (49.6)	0.22	− 0.29	0.95	1.18
Diabetes, n (%)	59 (27.8)	27 (22.7)	0.12	0.25	1.14	1.45
Antithrombotic drug use, n (%)	42 (19.8)	9 (7.6)	0.39	− 0.60	2.52	0.54
CVD, n (%)	46 (21.7)	14 (11.8)	0.27	− 0.57	1.63	0.62
Previous treatment, n (%)	49 (23.1)	2 (1.7)	0.69	− 0.49	10.71	0.57
Tumor size (mm), mean (SD)	21.2 (7.1)	25.6 (7.2)	− 0.62	− 0.40	0.95	1.45
R.E.N.A.L nephrometry score, mean (SD)	6.5 (1.7)	6.8 (1.7)	− 0.16	− 0.51	0.99	0.88

*PCA* percutaneous cryoablation, *RAPN* robot-assisted partial nephrectomy, *SD* standard deviation, *ASA* American Society of Anesthesiologists, *CCI* Charlson Comorbidity Index, *CVD* cardiovascular diseases, *SMD s*tandardized mean difference, *VR* variance ratio

If the IPWRA did not converge due to data limitations, we used Regression Adjustment (RA) as an alternative. The RA adjusts for confounding factors by including covariates in the regression model directly, thereby providing adjusted treatment effect estimates. Unlike the IPWRA, the RA lacks double robustness; however, it is computationally more stable and suitable as an alternative.

Similar comparisons were performed between the PCA (with and without TAE) and RAPN groups. Complication rates were compared using the chi-square test. The total cost and procedural costs for cases using two and three cryoprobes in the PCA were compared using Welch’s test. All analyses were conducted using Stata 18/MP4 (Stata Corp, College Station, TX, USA), and a *P* value of < 0.05 was considered statistically significant. Since this was an exploratory study, no adjustments were made for multiple comparisons [22]. A standardized mean difference (SMD) below 0.10 and a variance ratio (VR) between 4/5 and 5/4 were used to confirm covariate balance assumptions [23, 24].

## Results

### Patients

**Fig. 1:**
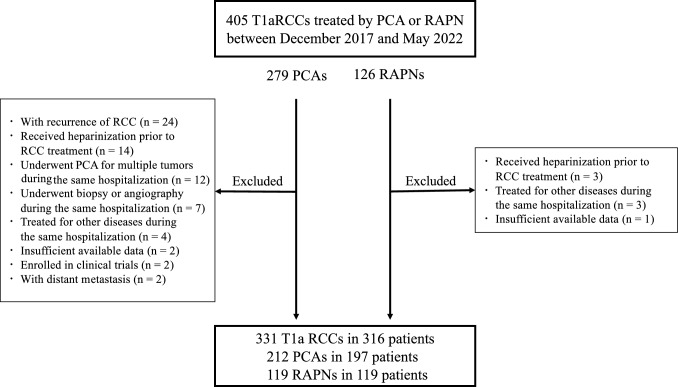
Study flow chart of clinical T1a RCC

During the study period, a total of 405 T1a RCCs were treated, comprising 279 PCAs and 126 RAPNs. Of these, 331 RCCs (212 PCAs and 119 RAPNs) in 316 patients met the inclusion criteria (Fig. 1). In the PCA group, 212 sessions were performed on 197 patients. Of these, 155 RCCs were treated with a combination of PCA and TAE, while 57 RCCs were treated with PCA alone. In the RAPN group, 119 sessions were performed on 119 patients.

### Clinical data

PCA patients were older and had slightly lower BMI. They also had higher ASA scores and CCI. Additionally, they were more likely to have a history of previous RCC treatment, CVD, and antithrombotic drug use (Table 2). Additionally, 8% of the PCA patients had a single kidney, and 16.5% had hereditary RCC, whereas none of the RAPN patients presented with these conditions.

The SMD values exceeded 0.1 for many variables before and after IPW, and VR values exceeded the 4/5–5/4 range, indicating that covariate balance could not be achieved with IPW. Therefore, a doubly robust method was employed. When both SMD and VR exceeded the criteria, a difference between the two groups was concluded.

PCA was associated with significantly shorter procedure time and hospitalization duration than was RAPN. Moreover, hospitalization duration for patients without TAE was shorter than for those with TAE (Table 3).

**Table 3:** Hospitalization duration and procedure time

Characteristic	RAPN (n = 119)				PCA					
		PCA total (n = 212)			PCA with TAE (n = 155)			PCA without TAE (n = 57)		
	Mean(SD)	Mean(SD)	Differences (95% CI)^a^	*P* value*	Mean(SD)	Differences (95% CI)^a^	*P* value*	Mean(SD)	Differences (95% CI)^a^	*P* value*
Hospitalization duration, mean (SD) [days]	9.88 (5.46)	6.96 (1.75)	− 2.2(−2.7, − 1.7)	< 0.001	7.34 (1.79)	− 1.9(− 2.5, −1.4)	< 0.001	5.95 (1.16)	− 3.1(− 3.7, − 2.5)	< 0.001
Procedure time, mean (SD) [min]	175.41 (42.16)	130.73 (40.94)	− 37.9(− 46.6, − 29.2)	< 0.001						

*PCA* Percutaneous cryoablation; *RAPN* robot-assisted partial nephrectomy

*P value: IPWRA converge data

^a^PCA minus RAPN amount

Major complications occurred in three PCA and four RAPN patients (Table 4). One RAPN patient had a grade IV complication of hemorrhagic shock due to retroperitoneal hemorrhage, necessitating blood transfusion, emergency TAE, ICU admission, and eventual open nephrectomy. The major complication rate was lower in the PCA group than in the RAPN group (1.4 vs. 3.4%); however, the difference was not statistically significant (*P* = 0.238).

**Table 4:** Major complication of each treatment

	PCA (n = 212)	RAPN (n = 119)	
Ureteral injury (Grade IIIa)		1	
Ureteral stenosis (Grade IIIa)	1		
Pneumothorax (Grade IIIa)		1	
Hematuria (Grade IIIa)	2	1	
Retroperitoneal hemorrhage (Grade IV)		1	
Total (%)	3 (1.4)	4 (3.4)	*P* = 0.238*

*PCA* percutaneous cryoablation; *RAPN* robot-assisted partial nephrectomy

*P value calculated by Chi-squared test

### Cost data

Cost data details are summarized in Table 5. In raw data analysis, the total cost of PCA was slightly lower than that of RAPN. PCA with TAE had a higher total cost than that of RAPN; however, the cost of PCA without TAE was lower. Using three cryoprobes in PCA significantly increased the total cost compared to using two cryoprobes (768,000 ± 32,000 vs. 586,000 ± 81,000 yen; difference: 182,000 yen, 95% CI 173,000–191,000 yen,* P* < 0.001). After RA adjustment, PCA’s total cost was significantly higher than that of RAPN. PCA with TAE had a significantly higher cost than that of RAPN, whereas PCA without TAE had a slightly lower cost than that of RAPN.

**Table 5:** Summary of Cost Data (Thousand JPY*)

	RAPN (n=119)	PCA total (n=212)					PCA with TAE (n=155)					PCA without TAE (n=57)				
			Raw		IPWRA			Raw		IPWRA			Raw		IPWRA	
	Mean (SD) [range]	Mean (SD) [range]	vs RAPN (95%CI)^b^	*P* value	vs RAPN (95%CI)^b^	*P* value	Mean (SD) [range]	vs RAPN (95%CI)^b^	*P* value	vs RAPN (95%CI)^b^	*P* value	Mean (SD) [range]	vs RAPN (95%CI)^b^	*P* value	vs RAPN (95%CI)^b^	*P* value
Total income	1404 (1105) [1120 to 13,324]	1030(173) [710 to 1579]	− 374 (− 573, − 175)	< 0.001	− 244 (− 325, − 164)	< 0.001^a^	1120 (100) [976 to 1579]	− 284 (− 482, − 86)	0.005	− 163 (− 244, − 82)	< 0.001^a^	786 (55) [710 to 950]	− 618 (− 816, − 419)	< 0.001	− 481 (− 516, − 446)	< 0.001
Total cost	1155 (1116) [855 to 13,176]	1123 (149) [751 to 1691]	− 32 (− 233, 169)	0.755	114 (31, 197)	0.007^a^	1181 (117) [916 to 1691]	27 (− 174, 227)	0.795	164 (125, 204)	< 0.001	964 (103) [751 to 1207]	− 191 (− 392, 10)	0.063	− 20 (− 109, 69)	0.660^a^
Total income and Expenditure balance	249 (63) [96 to 373]	− 93 (105) [− 330 to + 256]	− 342 (− 360, − 324)	< 0.001	− 358 (− 377, − 338)	< 0.001	− 62 (87) [− 330 to + 256]	− 311 (− 328, − 293)	< 0.001	− 320 (− 339, − 301)	< 0.001	−178 (102) [− 311 to + 153]	− 427 (− 455, − 398)	< 0.001	− 469 (− 500, − 438)	< 0.001
Procedural income^c^	923 (31) [868 to 1014]	549 (57) [529 to 763]	− 374 (− 384, − 365)	< 0.001	− 369 (− 381, − 358)	< 0.001										
Procedural cost^c^	693 (69) [600 to 896]	739 (77) [568 to 1006]	46 (30, 62)	< 0.001	72 (55, 88)	< 0.001^a^										
Procedural Income expenditure balance^c^	231 (63) [78 to 355]	−189 (92) [− 477 to + 153]	− 420 (− 437, − 403)	< 0.001	− 439 (− 457, − 422)	< 0.001										

*PCA* percutaneous cryoablation, *RAPN* robot-assisted partial nephrectomy, *SD* standard deviation, *95%CI* confidence interval, *JPY* Japanese yen

^*^The unit of notation is thousand JPY

^a^Since the IPWRA calculation did not converge, it was calculated using RA instead

^b^PCA minus RAPN amount

^c^“Procedural” means RAPN or PCA

The procedural cost of PCA was significantly higher than that of RAPN. Using three cryoprobes also significantly increased the procedural cost compared to two cryoprobes. After RA adjustment, PCA’s procedural cost remained significantly higher than that of RAPN.

PCA had a significantly worse total income-expenditure balance than that of RAPN. Both PCA with and without TAE showed a significantly worse total income-expenditure balance than that of RAPN. After IPWRA adjustment, PCA’s balance remained significantly worse than that of RAPN.

The procedural income-expenditure balance was also significantly worse for PCA than for RAPN. This result was consistent after IPWRA adjustment.

### Cost-effectiveness analysis

Pierorazio et al.’s systematic review and meta-analysis [20] showed an effectiveness of 90.1% for thermal ablation and 85.1% for partial nephrectomy, reflecting a 5% incremental effectiveness. As a result, calculating the ICER with the current costs yielded − 640,000 yen, indicating that PCA was the preferred option (Table 6).

**Table 6:** Cost-effectiveness results

Treatment	Mean Cost (JPY)	Effectiveness*[20]	Mean Incremental cost (JPY)	Incremental effectiveness	Incremental cost-effectiveness
RPN	1,155,000	0.85	–	–	Dominated
PCA	1,123,000	0.9	− 32,000	0.05	− 640,000

*PCA* Percutaneous cryoablation, *RPN* robot-assisted partial nephrectomy, *JPY* Japanese yen, Incremental cost: difference between cost of PCA and RPN, Incremental effectiveness: difference between effectiveness of PCA and RPN, Incremental cost-effectiveness: ratio between incremental cost and incremental effectiveness

*Effectiveness was evaluated based on the frequency of complications reported in systematic review [20]

## Discussion

The procedural cost of PCAwas higher than that of RAPN. Despite the higher procedural costs and the inclusion of less favorable patients, the unadjusted total costs of PCA were slightly lower than those of RAPN, likely due to the high expense of treating one RAPN patient with grade IV complications. Determining the true cost is complex because of the intricacies in hospital accounting and the number of stakeholders involved in each treatment [16]. Therefore, our findings, which were obtained under specific circumstances at a single institution, should be interpreted with caution. Nevertheless, they may provide valuable insights into RCC treatment from a cost perspective, as such data has not been previously provided in Japan.

Several studies have compared the perioperative, oncological, and renal functional outcomes between PCA and RAPN for T1 RCC [10–14]. Major complication rates were similar between the groups. While overall survival did not significantly differ, recurrence-free survival showed some variation: in some studies, it was not significantly different [10, 11], while in others, recurrence-free survival was significantly higher with RAPN than with PCA [12–14]. Renal function was either equally preserved or significantly improved after PCA treatment. PCA provides several advantages: it is less invasive, can be performed percutaneously under local anesthesia, involves short hospital stays, is repeatable, better preserves renal function, and is more suitable for high-risk patients. In Japan, the cost is unlikely to be a major factor in treatment selection, as the national health insurance system is comprehensive, ensuring that patients do not incur more than a predefined amount.

Cost comparisons between PCA and RAPN for T1aRCC have been reported in the United States (37 PCAs and 102 RAPNs) [15] and Brazil (59 PCAs and 63 RAPNs) [16]. In both countries, the cost of PCA was significantly lower than that of RAPN (*P* < 0.001): $6803 vs. $13,242 in the United States [15] and $12,435 vs. $19,399 in Brazil [16]. RAPN was 1.56 and 1.95 times more expensive than PCA in the United States and Brazil, respectively. Direct cost comparisons with other countries may not be appropriate because of the differences in healthcare systems and treatment protocols.

Japan’s DPC/PDPS employs a hybrid payment model: daily bundled payments cover hospitalization and tests, while fixed fees are paid for advanced surgical procedures such as RAPN and PCA.

In contrast, many countries, including the United States, use the Diagnosis-Related Group/Prospective Payment System, which adopt case-based bundled payments that typically include surgical procedures. Additionally, in the U.S., Depending on the type of insurance enrolled and medical fields allow for private medical care, where doctor fees can be exceptionally high, contributing to rising healthcare costs (hospital payment). This significantly impacts hospital financial balance.

In countries where there have been past reports, PCA is typically performed under general anesthesia, whereas in Japan, it is usually performed under local anesthesia. Additionally, the number of cryoprobes was one or two [16], and the hospital stay was short (1.1–2.2 days) [15, 16], which likely contributed to the low PCA costs.

Previous studies have emphasized the cost-effectiveness of PCA. For instance, Garcia RG et al. reported that PCA demonstrated a 5% higher incremental effectiveness than RAPN, resulting in cost savings [16]. Similarly, Wu et al. reported that PCA was more cost-effective than partial nephrectomy in 84.78% of the Monte Carlo simulations of T1a RCC [17].

For T1a RCC, resection is the first-choice treatment. Some PCA patients were medically unfit for RAPN. Consequently, PCA patients tended to have worse conditions than did RAPN patients, such as older age, higher ASA scores, higher CCI, antithrombotic drug use, presence of CVDs, history of previous RCC treatment, and single kidney status. It is also possible that if these patients had undergone RAPN, they might have experienced longer hospitalizations, potentially leading to higher costs.

Several factors affected costs, including the anesthesia method, with PCA typically performed under local anesthesia and RAPN requiring general anesthesia. Additionally, in some facilities, biopsies may be performed during the same hospitalization period as PCA, potentially increasing both hospitalization duration and costs. To ensure accurate comparisons, this study excluded cases where biopsies were performed during the same hospitalization and also excluded the fee for pathological diagnosis in RAPN. The onset of complications significantly affects the total cost, particularly when additional treatment is necessary. In the case of PCA, performing TAE before ablation affected both costs and income. The use of TAE slightly improved the negative income-expenditure balance and may have reduced the total costs by lowering the risk of bleeding complications during cryoablation. As expected from the economic evaluation of advanced technologies, individual procedural costs had the greatest influence on cost-effectiveness ratios, primarily due to the high proportional cost of the materials (cryoprobes and robotic clamps) [16].

In this study, cryoprobes and robotic clamps, which are not reimbursed, likely had the most remarkable impact on procedural costs. The procedural cost of PCA increases with tumor size due to the use of additional cryoprobes, whereas the cost of RAPN remains constant. Consequently, the cost difference between RAPN and PCA became more substantial for larger tumors. To ensure that PCA, which has significant demand, can be performed without financial loss for the hospital, it is necessary to either charge separately for the cryoprobes or increase the insurance points.

This single-institution, retrospective study has several limitations. First, it was challenging to fully account for all costs, including operating room use, ICU stay, and labor costs outside the procedure. Second, individual costs may differ among institutions. Third, imaging costs, such as CT scans performed for complication management, were excluded from the analysis. Fourthly, this study focused on short-term cost and cost-effectiveness comparison; further investigation is required for indirect costs, such as the time required for patients to return to work, productivity loss, long-term tumor control, and overall cost-effectiveness. Fifth, TAE may impact not only hospital finances but also treatment effectiveness; however, this was not examined in the present study. Sixth,, there is a fundamental difference in that PCA costs vary depending on tumor size, while RAPN remains unchanged. Finally, the results are based on Japan’s national insurance system and may not be directly applicable to other countries with different healthcare systems and cost structures. In the two countries cited [15, 16], both PCA and RAPN were more expensive than in Japan. The propensity score adjustment was insufficient; however, balancing was achieved with RA.

In conclusion, for high- risk patients, PCA demonstrated a safer option with shorter hospitalization duration than RAPN. Although PCA was more cost-effective in terms of total hospitalization costs, its higher procedural cost and unfavorable income-expenditure balance require careful evaluation, especially for large tumors that require three or more needles. Our findings provide valuable insights into RCC treatment from a cost perspective, as such data have not been previously provided in Japan.
